# Aortic valve replacement via right anterolateral thoracotomy in the case of a patient with extreme mediastinal right-shift following pneumonectomy

**DOI:** 10.1186/1749-8090-8-20

**Published:** 2013-01-25

**Authors:** Mathias Wilhelmi, Thomas Rodt, Issam Ismail, Axel Haverich

**Affiliations:** 1 Division for Cardiac, Thoracic, Transplantation, and Vascular Surgery, Hannover Medical School, Carl-Neuberg-Str. 1, Hannover, 30625, Germany; 2 Diagnostic and Interventional Radiology, Hannover Medical School, Carl-Neuberg-Str. 1, Hannover, 30625, Germany

**Keywords:** Pneumonectomy, Aortic valve, Thoracotomy, Minimally invasive surgical procedures

## Abstract

We report on the case of a 68-year-old male patient with the history of right pneumonectomy due to bronchial carcinoma, who was referred for aortic valve replacement due to severe calcified aortic stenosis. Pre-operative chest X-ray and computed tomography (CT) revealed an unusually pronounced mediastinal shift to the right. Despite this unusual anatomy, we decided to perform surgery using the right anterolateral thoracotomy following thorough pre-operative planning using 3D-volume rendering of the CT data-set. This approach yielded excellent exposure of the aortic root and the ascending aorta, respectively. Following an uneventful operative and post-operative course the patient could be discharged on post-OP day 6.

Although only occasionally described for aortic valve replacement a right anterolateral thoracotomy may represent a valuable surgical approach, particular in patients with unusual anatomy, e.g. a mediastinal right-shift. However, thorough pre-operative planning, i.e. using visualization and planning techniques such as 3D-volume rendering should be mandatory as it provides information crucial to facilitate surgical steps and thus, may help avoid severe surgical complications.

## Background

Aortic valve replacement is a commonly known and well-established surgical procedure, often performed by young cardiac surgeons. Although minimally invasive procedures are increasingly performed in this as well as in other regions, the most commonly used surgical approach to the aortic valve is still the median sternotomy. Here we report on an aortic valve replacement via right anterolateral thoracotomy in the uncommon case of a patient with severe aortic valve stenosis and extreme mediastinal right-shift due to previous pneumonectomy.

## Case presentation

**Figure 1:**
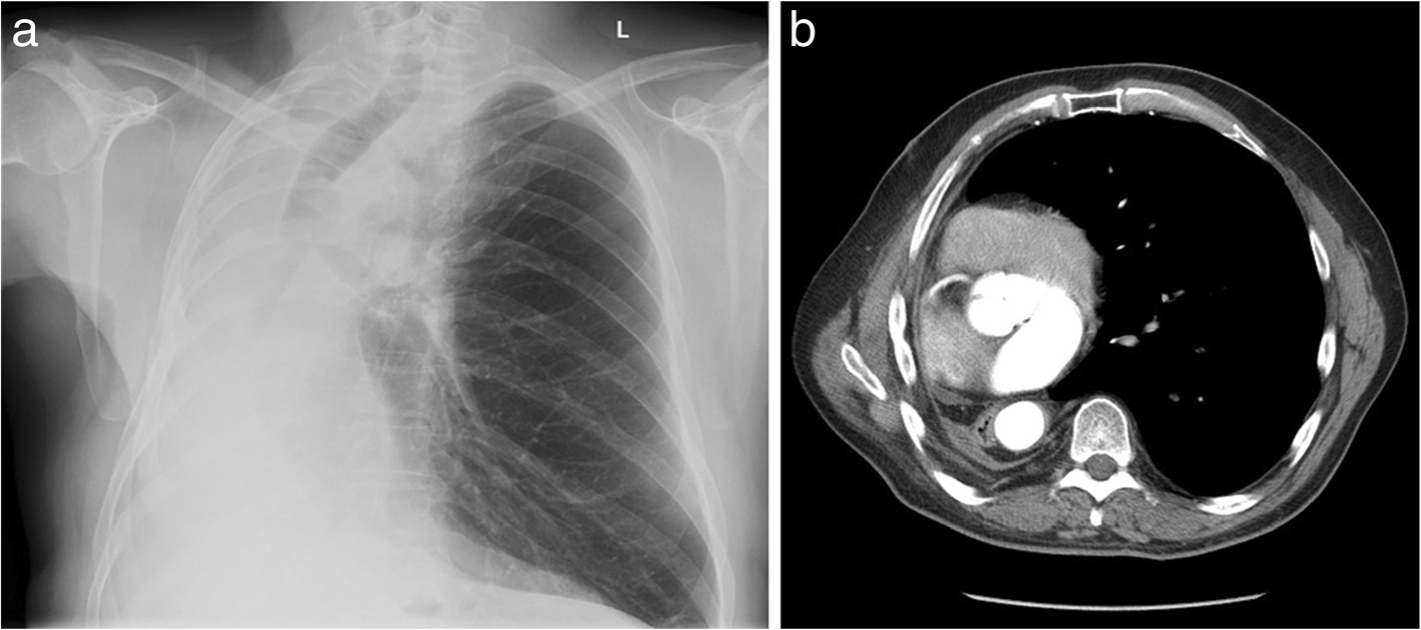
**Pre-operative chest X-ray (Figure **1**a) and computed tomography (Figure **1**b) revealing complete mediastinal and cardiac right-shift.**

**Figure 2:**
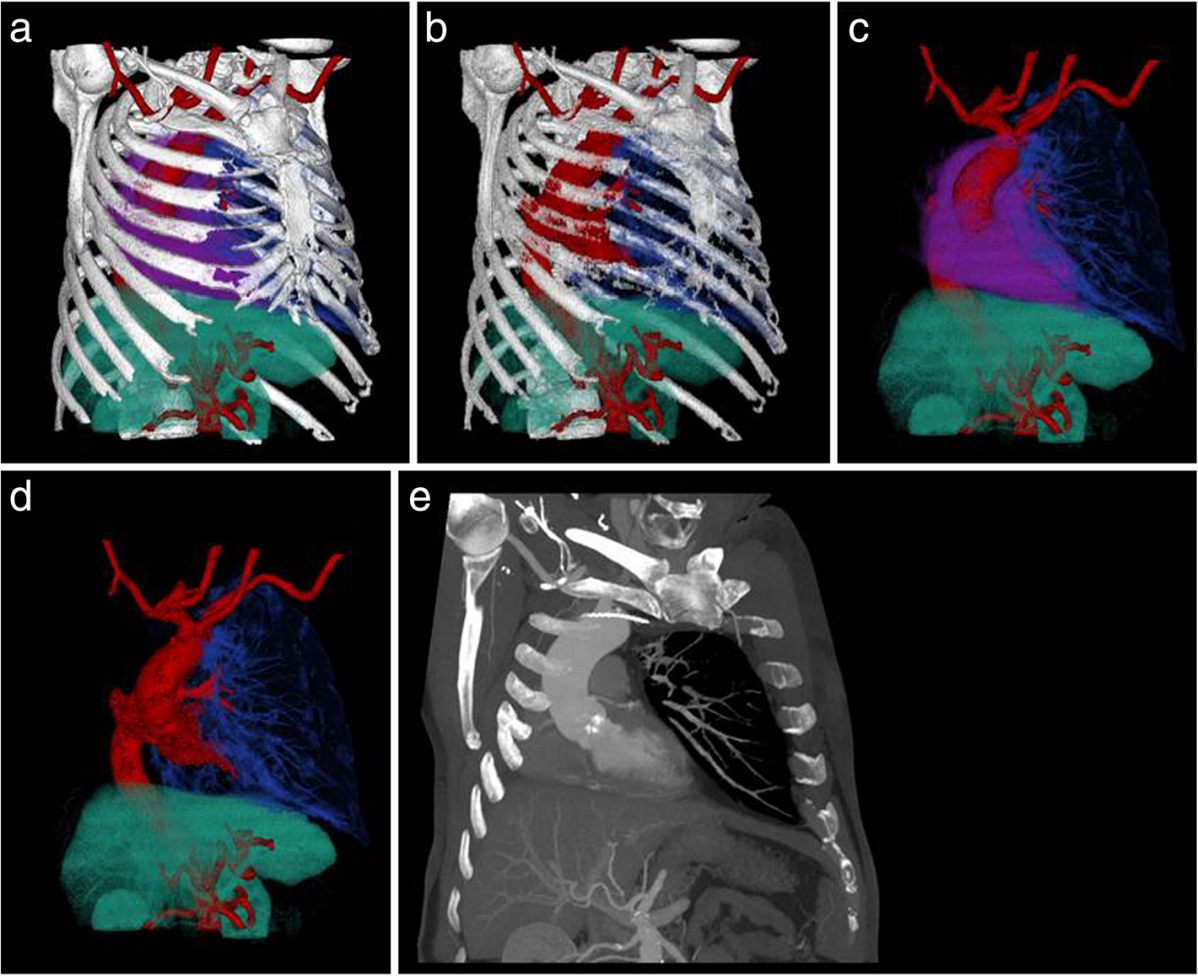
**2a-d: 3D-Volume Rendering (approximately 20° RAO and 10° superior view) for surgical planning. **Segmentation volumes of bony chest structures, heart, aorta and aortic braches and abdominal organs were generated using threshholding and semiautomated segmentation techniques. Image characteristics (such as colour or transparency) of the individual anatomic structures could then be deefined separately allowing interactive 3D-visualization of the spatial relationship of the anatomical structures. **2e:** 50 mm maximum intensity projection slab (slab orientation approximately 20° RAO and 10° superior corresponding to Figure 2**a**-**d**) of the aortic arch showing the calcification of the aortic valve. No significant calcifications of the aortic arch.

A 68-year-old male patient with a severe calcified and stenosed aortic valve (valvular area 0.68 cm^2^; aortic stenosis III°) was referred for aortic valve replacement. History revealed a right pneumonectomy due to bronchial carcinoma eight years ago via right thoracotomy. The patient`s pulmonary function was modest (FEV1: 56%; FVC: 58%; GOLD 2° = modest obstruction), left ventricular function light to moderately restricted (LVEF 60%). Pre-operative chest X-ray and computed tomography revealed an unusually pronounced mediastinal shift to the right (Figure 1a and b). Despite presumptive severe adhesions following right pneumonectomy and following further pre-operative planning using 3D-volume rendering of the CT-data set (Figure 2a-f), it was decided to perform surgery via minimally invasive right anterolateral thoracotomy.

The patient was in a left semi-recumbent position and the anterolateral thoracotomy was placed along the submamarian margin. Despite some tissue adhesions this approach yielded excellent exposure of the aortic root and the ascending aorta, respectively. Cardiopulmonary bypass was established with arterial and venous cannulae in the right groin. The left ventricle was vented via the right superior pulmonary vein. Body temperature was cooled down to 34°C and ventricular fibrillation was induced electrically. Following a hockey stick-like incision of the extremely thin and fragile appearing aorta, the severe calcified aortic valve was resected and replaced by a biological valve (25 mm perimount magna Ease, Edwards Life sciences, Irvene, CA, USA).

Following an uneventful intra- (cross-clamping: 36 min; total perfusion time: 69 min; re-perfusion time: 26 min) and post-operative course, the patient could be discharged on post-OP day 6.

Apart from complete median incisions, which still represent the most commonly used approach in cardiac surgery, less invasive approaches are increasingly performed, i.e. to shorten the length of hospital stay, to lower treatment expenses as well as to reduce overall postoperative morbidity and mortality and last not least to provide better cosmetic results [1-3]. Surgical approaches to the aortic root/valve beside complete median sternotomy [4-8] comprise (T-shaped or transverse) ministernotomies [9] and more or less minimally invasive right- and left-sided thoracotomies. However, only very few studies have reported on the outcome following aortic valve replacement via right thoracotomy so far. Glauber et al. presented a larger series of “standard“ patients *(prior cardiac interventions, right-sided pleuritis and aortic root dilatation etc. were excluded)*, who underwent aortic valve replacement this way. They described low rates of in-hospital mortality (1.5%) and post-operative complications and thus, regarded the procedure to be safe. Even though they observed prolonged cross clamping- and cardiopulmonary bypass times as well as higher incidences of reexplorations for bleeding (3-8%), low rates of renal failure, atrial fibrillation, postoperative stroke and short postoperative recovery argues for this approach – at least under “standard“conditions [10]. Main reasons against an (right) anterolateral thoracatomy are reported higher incidences of intraoperative stroke mainly due to retrograde perfusion in the case of femoral cannulation as well as the limited possibility to deair the left ventricle. Other possible limitations comprise respiratory complications and insufficient myocardial protection [6,11].

Indeed, proper surgical exposure is crucial for every surgical intervention regardless of it´s anatomic location. However, it is in the nature of minimally invasive approaches that they provide only limited space and thus, surgical view. Therefore, thorough pre-operative planning is mandatory, especially in the case of minimally invasive- and/or re-do procedures. In the case presented here pre-operative planning using 3D-visualization of CT-data provided important information regarding anatomic dimensions and sizes and allowed for exclusion of mobile plaques within the aorta and the iliac/femoral vasculature. Thus, these data provided important information to lower the above mentioned risk factors of a minimally invasive approach and led us to the consideration that an anterolateral approach might be not only feasible but also reasonable in this particular situation of mediastinal right-shift.

## Conclusion

A right anterolateral thoracotomy may represent a valuable surgical approach for aortic valve replacement in patients with distinct anatomic conditions or following previous surgical interventions, e.g. a pneumonectomy. However, thorough pre-operative planning using imaging techniques such as 3D-volume rendering should be mandatory as it provides essential information to plan, simulate and finally facilitate surgical steps and thus, may help to avoid severe surgical complications.

## Consent

Written informed consent was obtained from the patient for publication of this case report and accompanying images. A copy of the written consent is available for review by the Editor-in-Chief of this journal.

## Competing interests

The authors declare that they have no competing interests.

## Authors’ contributions

This manuscript is conceived, drafted and finally written by MW. TR was responsible of the acquisition of radiological data and together with II interpreted the data prior to the surgical procedure, performed by II. After critical revision of the resulting manuscript by AH and II. All authors have read and approved the final manuscript.
